# Comparing the effects of laparoscopic radical surgery and traditional open surgery on short-term efficacy and long-term survival in patients with colorectal cancer

**DOI:** 10.3389/fsurg.2025.1729392

**Published:** 2026-01-30

**Authors:** Yanhong Lin, Jie Ling, Chuting Liao, Xiangjun Wang, Junfeng Yin

**Affiliations:** 1 School of Medical Nursing, Minxi Vocational & Technical College, Longyan, Fujian, China; 2 Department of General Surgery, The Affiliated Hospital of Yangzhou University, Yangzhou, Jiangsu, China

**Keywords:** colorectal cancer, immune function, laparoscopic radical surgery, stress response, survival rate

## Abstract

**Aim:**

This study aimed to compare the impacts of laparoscopic surgery (LS) and open surgery (OS) on the short-term efficacy and long-term survival in patients diagnosed with colorectal cancer (CRC).

**Methods:**

Sixty CRC patients who underwent LS at our hospital between January 2021 and January 2022 were enrolled as the LS group. Another 60 CRC patients who received OS during the same period at the same hospital were selected as the OS group. The study compared surgical parameters, postoperative recovery metrics, stress response indicators, inflammatory markers, immune function markers, the incidence of postoperative complications, quality of life assessments, and 3-year survival rates between the two cohorts.

**Results:**

The LS group exhibited a longer surgical duration but had shorter surgical incisions and less intraoperative blood loss compared to the OS group (*P* < 0.01). The number of lymph nodes dissected was similar in both groups (*P* > 0.05). The LS group also demonstrated quicker recovery, with shorter times to anal gas expulsion, defecation, oral intake, and activity, as well as a reduced hospital stay (*P* < 0.01). On the third day post-surgery, the study group showed lower levels of cortisol, epinephrine, and norepinephrine (*P* < 0.05), along with decreased levels of IL-6, TNF-α, and CRP (*P* < 0.05). Conversely, the study group had higher levels of CD3^+^, CD4^+^, and CD4^+^/CD8^+^ on the third day after surgery (*P* < 0.05). The overall incidence of postoperative complications was lower in the study group (*P* < 0.05). Twelve months post-surgery, both groups showed significant improvements in the Gastrointestinal Quality of Life Index (GIQLI) scores, with the study group outperforming the OS group (*P* < 0.05). Kaplan–Meier analysis revealed a 3-year survival rate of 81.67% in the study group vs. 80.00% in the OS group, with no statistically significant difference (*P* = 0.833).

**Conclusion:**

LS for CRC patients is highly effective, alleviating inflammatory and immune stress responses in patients, lowering the incidence of postoperative complications, improving the quality of life of patients, and having a long-term efficacy comparable to OS.

## Background

Colorectal cancer (CRC) ranks among the most prevalent malignant neoplasms affecting the digestive system (1). Data sourced from the GLOBOCAN database indicate that in 2020 alone, there were an estimated 1.9 million-plus newly diagnosed cases of CRC worldwide, accompanied by approximately 930,000 fatalities attributed to the disease (2). Recently, with the change in people's dietary habits, the incidence of CRC in China has been on the rise, seriously threatening the life safety of the people (3).

Radical resection remains the cornerstone of curative treatment for CRC, and traditional open surgery (OS) has long been regarded as the standard approach because it offers a clear operative field and facilitates thorough tumor removal (4, 5). However, OS is associated with relatively large incisions, greater tissue trauma, and a more pronounced systemic stress response, which may delay postoperative recovery. With the development of minimally invasive techniques, laparoscopic surgery (LS) has become a widely accepted alternative to OS in CRC. Several clinical studies and meta-analyses have shown that, compared with OS, LS achieves comparable oncologic clearance while providing advantages such as reduced intraoperative blood loss, faster recovery of gastrointestinal function, shorter hospital stay, and a lower overall incidence of postoperative complications (6–10).

Beyond these short-term clinical outcomes, accumulating evidence indicates that LS induces a milder neuroendocrine stress response and a less intense systemic inflammatory reaction than OS, as reflected by lower postoperative levels of cortisol, epinephrine, norepinephrine, IL-6, TNF-α, and C-reactive protein (CRP) (6, 11, 12). At the same time, LS appears to better preserve cellular and humoral immunity, with higher postoperative CD3^+^, CD4^+^, and CD4^+^/CD8^+^ ratios and less suppression of immunoglobulin levels compared with OS (13, 14).

However, the extent to which these differences in stress, inflammatory, and immune responses translate into improved recovery, fewer complications, and long-term outcomes in routine clinical practice remains incompletely clarified. Therefore, this study was designed to compare LS and OS in patients with CRC by systematically evaluating perioperative recovery, stress indicators, inflammatory markers, immune function, postoperative complications, quality of life, and 3-year survival. We hypothesized that LS would attenuate postoperative stress and inflammatory responses, better preserve immune function, and thus improve short-term recovery while maintaining long-term survival comparable to that of OS.

## Methods

### Study design and included patients

This was a single-center, retrospective observational cohort study. ixty consecutive CRC patients who met the eligibility criteria and underwent LS at our hospital between January 2021 and January 2022 were enrolled as the LS group. During the same period, all consecutive patients who met the same eligibility criteria but were treated with conventional open surgery formed the OS group. The choice between LS and OS was made after a comprehensive preoperative evaluation and shared decision-making between the attending colorectal surgeon and the patient, in accordance with our institutional practice (e.g., considering tumor characteristics, comorbidities, previous abdominal surgery, and patient preference). All patients and their families signed the informed consent form. This study was approved by the Ethics Committee of our hospital.

Inclusion criteria: (1) All patients were diagnosed preoperatively through CT imaging or colonoscopy, and the diagnosis was confirmed by postoperative pathology as CRC; (2) All patients met the indications for surgical treatment; (3) With complete clinical and follow-up data. All patients underwent routine preoperative staging with contrast-enhanced abdominal and pelvic CT, together with chest CT or chest x-ray; when indicated, additional imaging such as PET-CT or MRI was performed.

Exclusion criteria: (1) Non-primary CRC; (2) Radiologically confirmed distant metastasis on the preoperative staging workup; (3) Severe diseases of the heart, lungs, liver, and kidneys; (4) With other malignant tumors.

### Surgical methods

All operations in both groups were performed by the same colorectal surgery team, consisting of senior attending surgeons with extensive experience in both laparoscopic and open colorectal cancer resections. A standardized perioperative management protocol was applied to all patients.

For patients with low rectal cancer, the choice between Dixon and Miles procedures was based on tumor distance from the anal verge, sphincter function, and continence status, and Miles abdominoperineal resection was reserved for very low tumors or markedly impaired sphincter function.

The surgical principles of the LS and OS were the same, strictly adhering to the no-tumor principle. For CRC surgeries, the principle of total mesocolic excision (TME) was always followed.

The OS group underwent traditional OS. The patients were placed in a supine position, and general anesthesia was administered through tracheal intubation. A 10–15 cm incision was made at the center of the umbilicus. The lesion was thoroughly examined, and the specific surgical plan was chosen based on the location and size of the tumor. The lesion was effectively separated from the surrounding tissues, and intestinal anastomosis was performed following the operation.

The LS group underwent LS. The LS was performed under general anesthesia. A puncture at the navel was used to establish the pneumoperitoneum. A 10-mm puncture was made at the navel as the observation hole. Puncture holes at the level of the anterior superior iliac spines on both sides and outside the transverse abdominal muscle served as the main operation ports, and another 5-mm puncture in the right lower abdomen was used as an auxiliary port. Titanium clips or polymer clips were used to clamp and disconnect the vessels at the root of the mesentery. The laparoscope was used to free the intestinal segment and the mesentery and to clear the root of the mesenteric vessels and nearby lymph nodes. For patients undergoing intestinal anastomosis after colonic resection, a small abdominal incision was made to exteriorize the right or sigmoid colon, and an extracorporeal colorectal anastomosis was performed. In this cohort, ileoanal anastomosis was reserved only for selected patients with very low rectal lesions in whom sphincter-preserving resection was feasible but a standard colorectal anastomosis was not appropriate. For all remaining patients undergoing rectal resection, a small abdominal incision was made to disconnect the bowel, and a stapler was introduced transanally to complete the colorectal anastomosis.

### Outcomes

Surgical indicators including surgical duration, length of surgical incision, intraoperative blood loss volume, as well as number of lymph node dissections.

Postoperative recovery indicators including the time for anal gas expulsion, the time for defecation, the time for eating, the time for activity and the length of hospital stay.

On the day before the operation and 3 days after the operation, 3 mL of peripheral venous blood was gathered respectively. The blood was centrifuged at 3,000 r/min for 10 min. The serum was then obtained. The levels of stress response indicators (cortisol, epinephrine and norepinephrine) and inflammatory response indicators (IL-6, TNF-α and CRP) were detected using an enzyme-linked immunosorbent assay. The changes in immune function indicators (CD3^+^, CD4^+^ and CD4^+^/CD8^+^) were determined using a flow cytometer.

Incidence of postoperative complications such as incision infection, anastomotic fistula, pulmonary infection, intestinal obstruction as well as subcutaneous emphysema in patients during the perioperative period.

The Gastrointestinal Quality of Life Index (GIQLI) was employed to evaluate the patients' quality of life before the surgery and 1 month, 6 months and 12 months following the surgery (15). This scale consisted of 5 dimensions: subjective symptoms, physical physiological function, psychological emotional status, social activities, and special disease conditions, involving 36 items. Each item scored 0–4 points, and the total score was 144 points. The higher the GIQLI score, the higher the quality of life of the patients. In this study, the GIQLI was administered as a self-reported questionnaire: patients completed the Chinese version independently during outpatient follow-up visits, with a trained nurse available to clarify any items when necessary. The nurses who collected and recorded the GIQLI questionnaires were not involved in the surgical procedures or perioperative decision-making, but they were not formally blinded to the type of surgery.

The postoperative survival status of the patients was followed up through outpatient visits and telephone calls. The starting point was the day of the surgery. The deadline was January 31, 2025. All patients were followed up for 3 years or until the patients experienced tumor-related death. Information on postoperative tumor recurrence (local or distant) was not collected in a standardized and complete manner during follow-up; therefore, tumor recurrence was not analyzed as a formal endpoint in this study, and overall survival was selected as the primary long-term oncological outcome. The 3-year survival rates of the two groups were compared.

### Statistical analysis

Data analysis was conducted by means of GraphPad Prism 10.0 statistical software. For continuous measurement data that followed a normal distribution or approximately did so, the mean ± standard deviation was used to represent them, and t-tests were employed for comparisons between groups; for continuous measurement data that did not conform to a normal distribution, the median (interquartile range) was used for representation, and Shapiro–Wilks rank sum tests were employed for comparisons between groups; for count data, percentages or composition ratios were used for representation, and *χ*^2^ tests were employed for comparisons between groups. The survival rates were determined through the application of the Kaplan–Meier method, and comparisons of survival rate disparities between the two groups were conducted using the Log-rank test. A statistically significant difference was defined as one with a *P*-value less than 0.05.

## Results

### Baseline data

No significant differences were seen in gender, age, tumor site, TNM stage, tumor resection method, tumor diameter as well as differentiation degree between the two groups (*P* > 0.05, Table 1), implying the baseline date of the two groups were comparable.

**Table 1:** Baseline data between the two groups.

Baseline data	OS group (*n* = 60)	LS group (*n* = 60)	*χ*^2^/*t* value	*P* value
Gender			0.306	0.579
Male	36 (60.00)	33 (55.00)		
Female	24 (40.00)	27 (45.00)		
Age (years)	64.27 ± 9.14	64.68 ± 9.56	0.240	0.810
Tumor site			0.376	0.539
Rectum	18 (30.00)	15 (25.00)		
Colon (including sigmoid)	42 (70.00)	45 (75.00)		
TNM stage			0.174	0.916
Stage I	23 (38.33)	21 (35.00)		
Stage II	30 (50.00)	31 (51.67)		
Stage III	7 (11.67)	8 (13.33)		
Tumor resection method				
Radical operation	34 (56.67)	37 (61.67)	0.407	0.815
Dixon operation	17 (28.33)	16 (26.67)		
Miles operation	9 (15.00)	7 (11.66)		
Tumor diameter (cm)	4.71 ± 1.89	4.78 ± 1.96	0.199	0.842
Differentiation degree			0.318	0.852
High differentiation	14 (23.33)	13 (21.67)		
Moderate differentiation	31 (51.67)	34 (56.67)		
Low differentiation	15 (25.00)	13 (21.66)		

### Surgical indicators

**Figure 1:**
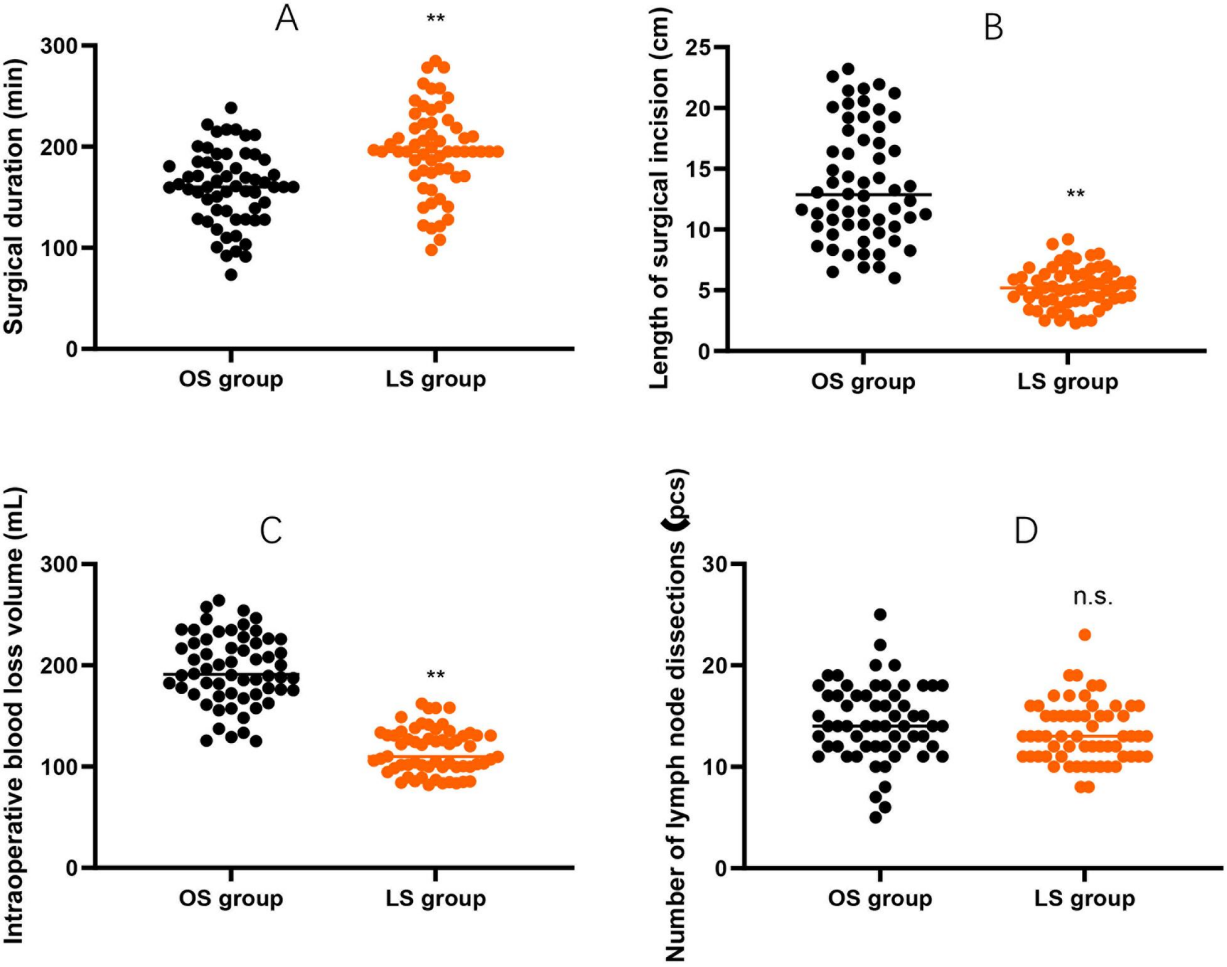
Surgical indicators between the two groups. **(A)** Surgical duration (min). **(B)** Length of surgical incision (cm). **(C)** Intraoperative blood loss volume (mL). **(D)** Number of lymph nodes dissected (pcs). Data are presented as mean ± SD. Between-group comparisons (LS vs. OS) were performed using independent-samples *t*-tests. **P < 0.01; n.s., no significant difference.

When compared to the OS group, the LS group exhibited a longer duration of surgery; however, they also had shorter surgical incisions and experienced a reduced volume of blood loss during the operation (*P* < 0.01). Notably, there was no statistical difference observed in the number of lymph nodes dissected between the two groups (*P* > 0.05, Figure 1).

### Postoperative recovery indicators

**Figure 2:**
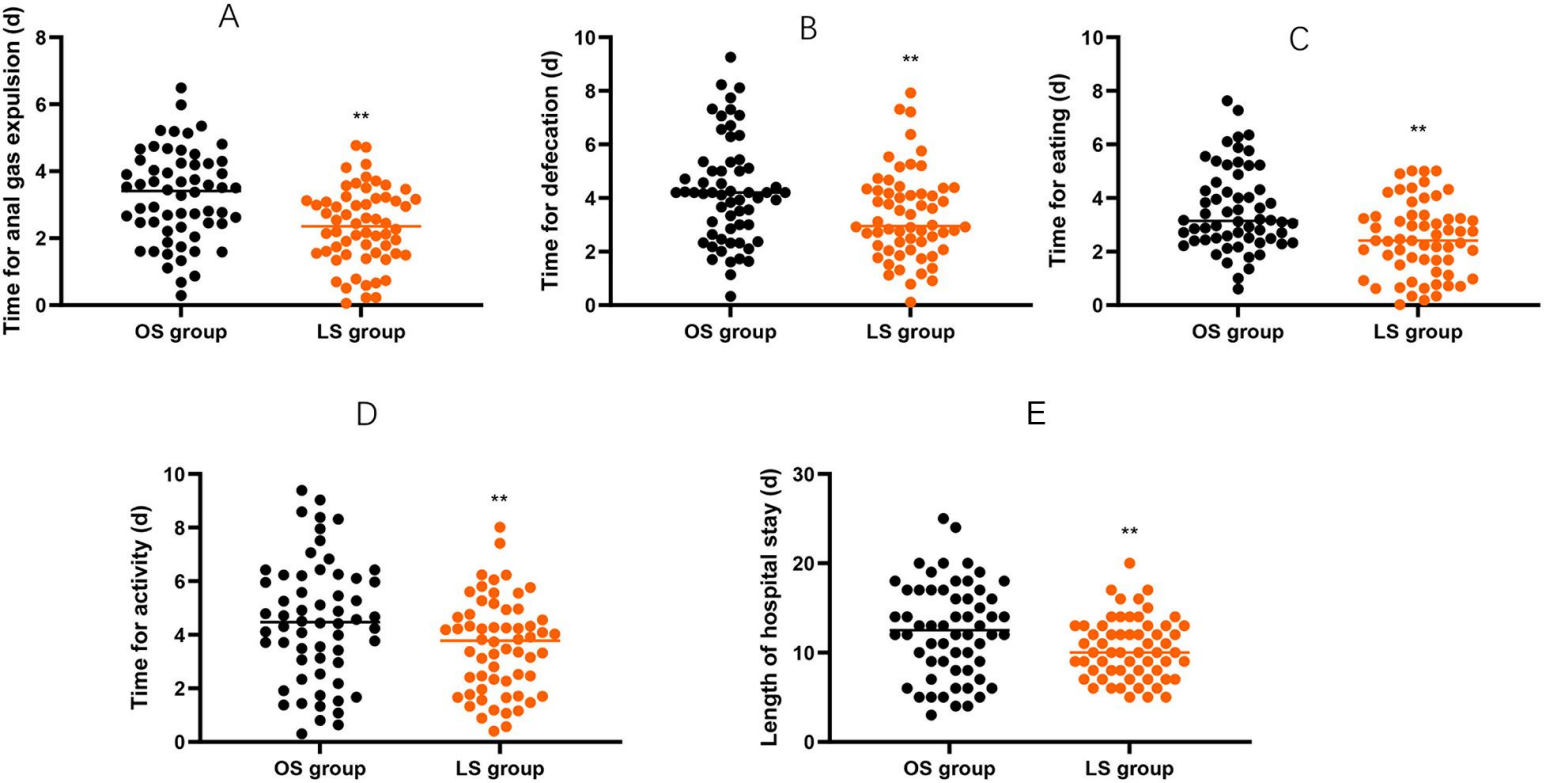
Postoperative recovery indicators between the two groups. **(A)** Time for anal gas expulsion (days). **(B)** Time for first defecation (days). **(C)** Time to oral intake (days). **(D)** Time to first out-of-bed activity (days). **(E)** Length of postoperative hospital stay (days). Data are presented as mean ± SD. Between-group comparisons at each endpoint were performed using independent-samples *t*-tests. **P < 0.01.

Compared with the OS group, the LS group exhibited shorter time for anal gas expulsion, shorter time for defecation, shorter time for eating, shorter time for activity and shorter length of hospital stay (*P* < 0.01, Figure 2).

### Stress response indicators

**Figure 3:**
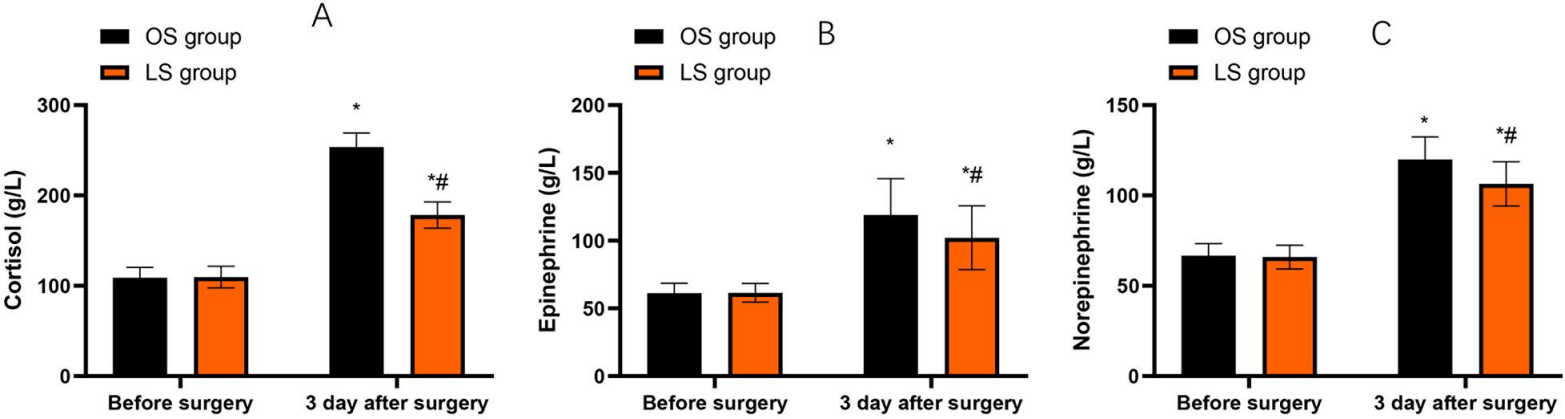
Stress response indicators before surgery and on postoperative day 3. **(A)** Cortisol levels (µg/L). **(B)** Epinephrine levels (µg/L). **(C)** Norepinephrine levels (µg/L). Bars represent mean ± SD for the OS and LS groups at each time point. Within-group changes (postoperative vs. preoperative) were assessed using paired *t*-tests, and between-group differences at the same time point were assessed using independent-samples *t*-tests. *P < 0.05 vs. before surgery; #*P* < 0.05 vs. OS group at the same time point.

Compared with before surgery, cortisol, epinephrine and norepinephrine levels in both groups were increased on the third day after the surgery (*P* < 0.05). Compared with the OS group, the LS group had lower levels of cortisol, epinephrine and norepinephrine on the third day following the surgery (*P* < 0.05, Figure 3).

### Inflammatory response indicators

**Figure 4:**
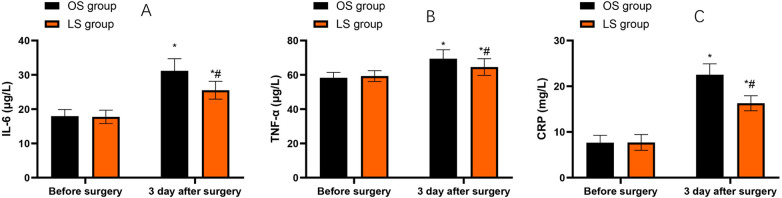
Inflammatory response indicators before surgery and on postoperative day 3. **(A)** IL-6 (µg/L). **(B)** TNF-α (µg/L). **(C)** CRP (mg/L). Bars represent mean ± SD for the OS and LS groups at each time point. Within-group changes (postoperative vs. preoperative) were assessed using paired *t*-tests, and between-group differences at the same time point were assessed using independent-samples *t*-tests. *P < 0.05 vs. before surgery; #*P* < 0.05 vs. OS group at the same time point.

Compared with before surgery, IL-6, TNF-α and CRP levels in both groups were increased on the third day after the surgery (*P* < 0.05). Compared with the OS group, the LS group had lower levels of the above inflammatory factors on the third day following the surgery (*P* < 0.05, Figure 4).

### Immune function indicators

**Figure 5:**
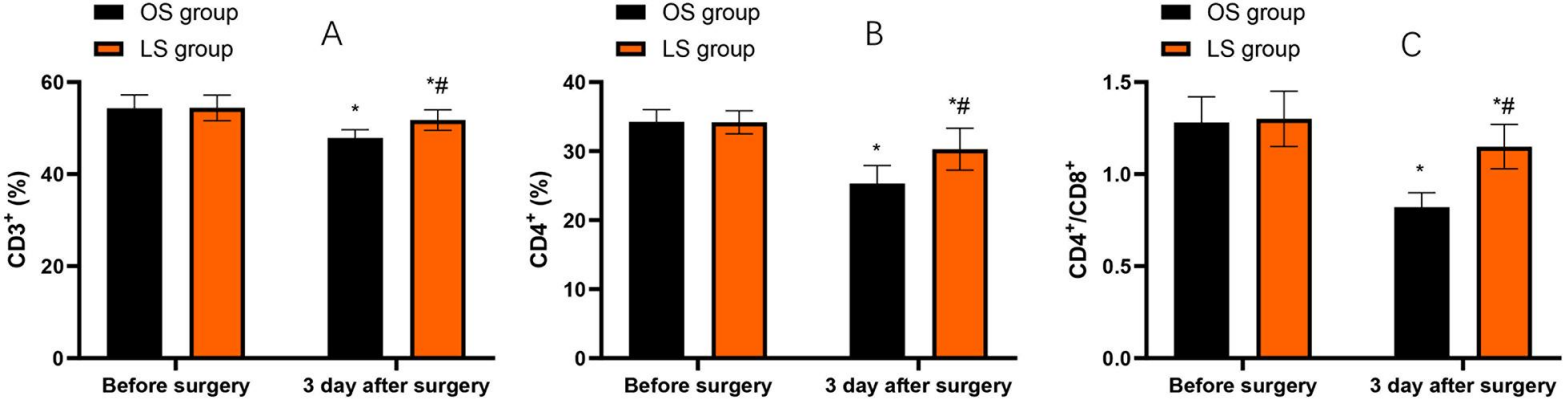
Immune function indicators before surgery and on postoperative day 3. **(A)** CD3^+^ (%). **(B)** CD4^+^ (%). **(C)** CD4^+^/CD8^+^ ratio. Bars represent mean ± SD for the OS and LS groups at each time point. Within-group changes (postoperative vs. preoperative) were assessed using paired *t*-tests, and between-group differences at the same time point were assessed using independent-samples *t*-tests. *P < 0.05 vs. before surgery; #*P* < 0.05 vs. OS group at the same time point.

Compared with before surgery, CD3^+^, CD4^+^ and CD4^+^/CD8^+^ levels in both groups were decreased on the third day after the surgery (*P* < 0.05). Compared with the OS group, the LS group had higher levels of the above immune function indicators on the third day following the surgery (*P* < 0.05, Figure 5).

### Incidence of postoperative complications

As shown in Table 2, in the LS group 2 patients (3.33%) developed incision infection, 2 (3.33%) anastomotic fistula, 1 (1.67%) intestinal obstruction, 1 (1.67%) pulmonary infection, and 1 (1.67%) subcutaneous emphysema, resulting in an overall complication rate of 7/60 (11.67%). In the OS group, 5 patients (8.33%) had incision infection, 4 (6.67%) anastomotic fistula, 3 (5.00%) intestinal obstruction, 2 (3.33%) pulmonary infection, and 2 (3.33%) subcutaneous emphysema, corresponding to a total complication rate of 16/60 (26.67%). The overall incidence of postoperative complications was significantly lower in the LS group than in the OS group (*χ*² = 4.356, *P* = 0.036).

**Table 2:** Incidence of postoperative complications between the two groups.

Groups	Cases	Incision infection	Anastomotic fistula	Intestinal obstruction	Pulmonary infection	Subcutaneous emphysema	Total incidence rate
LS group	60	2 (3.33)	2 (3.33)	1 (1.67)	1 (1.67)	1 (1.67)	7 (11.67)
OS group	60	5 (8.33)	4 (6.67)	3 (5.00)	2 (3.33)	2 (3.33)	16 (26.66)
*χ*^2^ value							4.356
*P* value							0.036

### Quality of life

**Figure 6:**
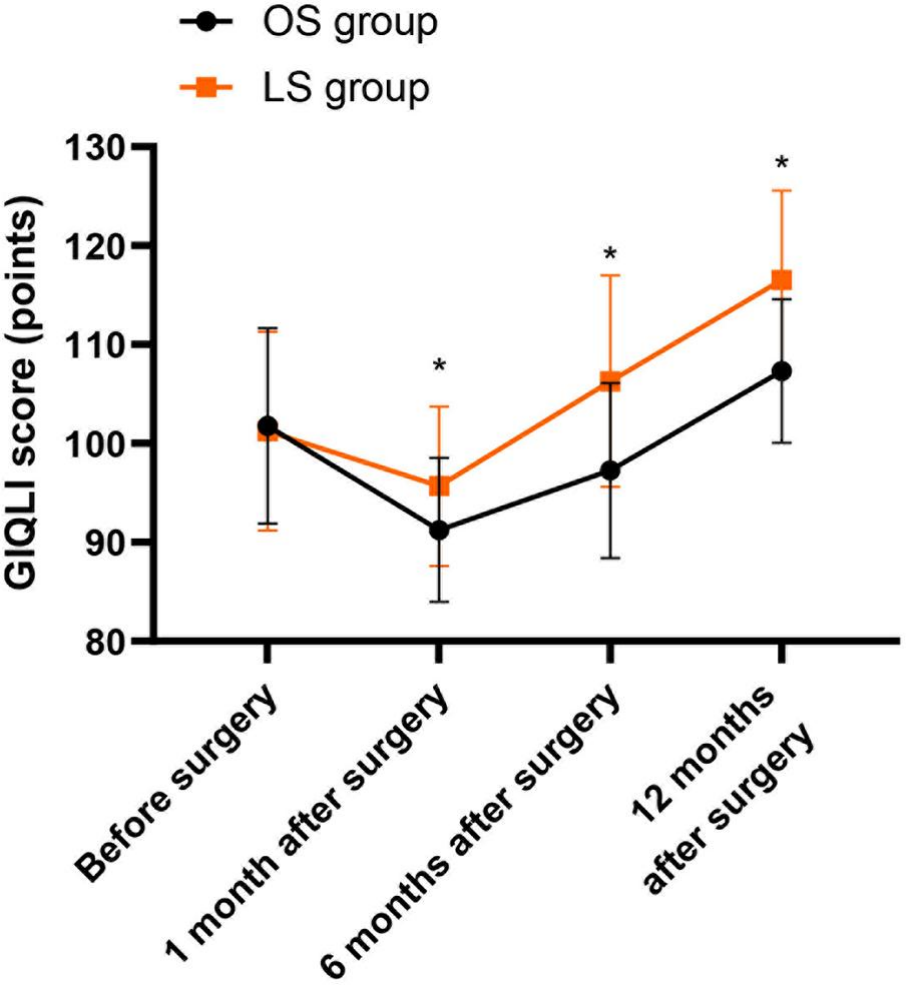
Gastrointestinal quality of life Index (GIQLI) scores before and after surgery. GIQLI scores in the OS and LS groups before surgery and at 1, 6, and 12 months after surgery. Data are presented as mean ± SD. Between-group comparisons at each time point were performed using independent-samples *t*-tests, and within-group comparisons with baseline were assessed using paired *t*-tests. *P < 0.05 vs. OS group at the same time point.

One month following the surgery, the GIQLI score of both groups showed a significant decline, but the LS group still performed better than the OS group (*P* < 0.05); six months following the surgery, the GIQLI score of both groups was significantly restored, and that of the LS group roughly returned to the preoperative level, while that of the OS group recovered relatively poorly (*P* < 0.05); twelve months following the surgery, the GIQLI score of both groups was significantly improved, and that of the LS group was better than the OS group (*P* < 0.05, Figure 6).

### Three-year survival rate

**Figure 7:**
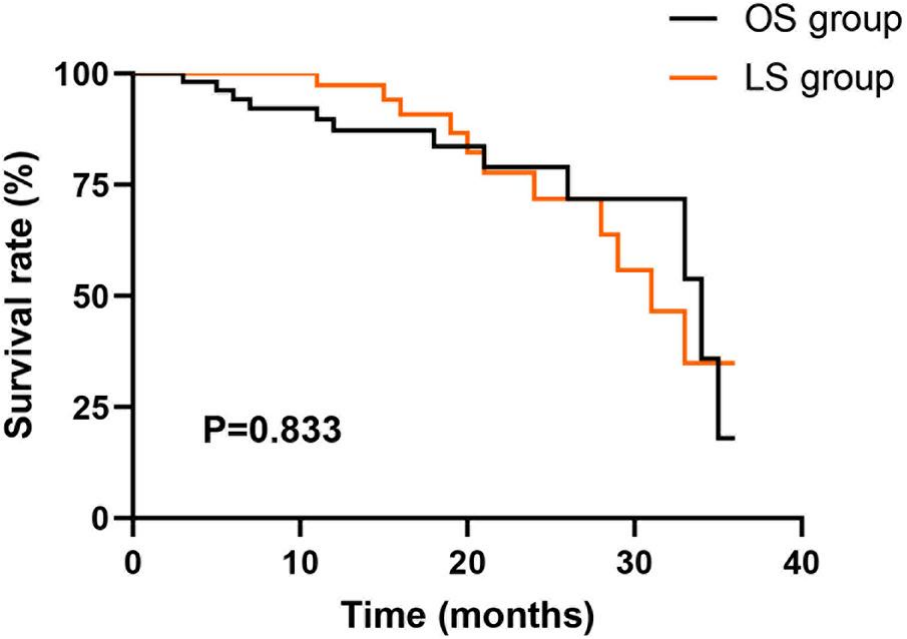
Three-year overall survival between the two groups. Kaplan–Meier overall survival curves for the OS and LS groups over a 36-month follow-up period. The *P*-value was calculated using the log-rank test.

According to the Kaplan–Meier analysis, the 3-year survival rate stood at 81.67% for the LS group, in contrast to 80.00% for the OS group. However, this difference had no statistical significance between the two cohorts (*P* = 0.833, Figure 7).

## Discussion

CRC ranks among the frequently encountered malignant neoplasms affecting the digestive tract (16). Recently, LS has developed rapidly and has also been applied to the treatment of CRC (17). However, there are still significant controversies regarding aspects such as surgical safety, tumor resection effectiveness, and long-term survival rates (8, 9). Therefore, this study compared and analyzed the clinical treatment effects, complications, as well as long-term survival conditions of CRC patients treated by the two surgical methods.

This study revealed that, compared with OS, LS had longer surgical duration, but it had the advantages of shorter length of surgical incision and less intraoperative blood loss volume. No statistical difference was seen in the count of lymph node dissections between the two cohorts. This finding implies that LS can obtain the same tumor resection effect as OS, and can more thoroughly remove the tumor and clear the metastatic lymph nodes, truly achieving the effect of “minimally invasive” treatment. Consistently, Suda et al. suggested that LS reduces intraoperative blood loss and postoperative hospital stay compared with OS in CRC patients (18).

This study compared the postoperative recovery conditions of two surgical methods. The results revealed that, patients underwent LS had shorter time for anal gas expulsion, shorter time for defecation, shorter time for eating, shorter time for activity and shorter length of hospital stay, indicating that LS can effectively reduce the recovery time of intestinal function for patients and significantly shorten their hospital stay. In addition, this LS revealed that the total incidence of postoperative complications in CRC patients undergoing LS was significantly reduced, suggesting that LS can minimize the trauma to the patient's body, thereby reducing the occurrence of related complications. In line with our findings, Li et al. suggested that LS serves as a secure and minimally invasive substitute for OS in patients with metachronous CRC. This approach leads to decreased estimated blood loss, quicker restoration of bowel function, and a shorter duration of postoperative hospitalization (10).

When the body is subjected to external stimuli such as surgical trauma, the concentrations of epinephrine and norepinephrine will significantly increase (19). Clinical studies have found that the changes in the concentrations of cortisol, epinephrine and norepinephrine during the early stage of the stress response are positively correlated with the severity of the patient's condition (20, 21). Thus, they can be used as an important indicator for evaluating the quality of the surgery. This research demonstrated that LS had a minimal impact on the stress response of CRC patients. This is because that that OS causes greater trauma and involves a wider range of tissue removal, which is more likely to trigger a stress response. In contrast, LS has less trauma and a smaller range of incision and traction, resulting in a relatively milder stress response. Likewise, He et al. discovered that LS of CRC can reduce surgical trauma, mitigate the inflammatory response, and alleviate pain stress induced by the surgical procedure (6).

All abdominal surgeries will cause varying degrees of impact on the abdominal function. Among them, the invasive operations during the surgery will lead to a significant inflammatory response in the body and have an immunosuppressive effect (11). IL-6 is an important cytokine that mediates immune regulation and inflammatory damage during the stress state, and it is also a major indicator for monitoring the severity of trauma (22). CRP is a representative acute-phase protein, which can significantly increase under stress conditions and is a constant elevated cytokine after tissue damage (12). TNF-α belongs to the most important and earliest mediator in the inflammatory response, which can activate lymphocytes and neutrophils, enhance vascular activity, as well as increase the synthesis and production of other immune cytokines (23). This study indicated that LS could effectively control the high expression of inflammatory cytokines in CRC patients after surgery compared to OS. Consistently, Bohne et al. conducted a systematic review and meta-analysis, which demonstrated that, compared to OS, LS for CRC patients result in a significantly less pronounced increase in postoperative levels of several proinflammatory markers (14).

Surgical trauma can trigger a strong stress response, and this stress response can lead to changes in the patient's immune function (24). T lymphocyte subsets are mainly important factors involved in the body's immune system and play a role in modulating immune responses and mediating immune functions (25). The results of our study indicated that LS caused less damage to the immune system of CRC patients compared to OS, which was beneficial for the recovery of the body's immune function. This may be related to factors such as the smaller incision, shorter operation time, and less postoperative inflammatory response of LS. Likewise, Bohne et al. put forward that LS might be associated with reduced suppression of cellular immunity and diminished inflammatory responses in CRC patients (13).

This study indicated that, one month after the operation, due to surgical trauma and postoperative complications, the quality of life of both groups decreased significantly, but the LS group still performed better than the OS group. As time went on, the influence of the surgery became weaker and weaker. Six months following the surgery, the quality of life of both groups significantly recovered, and that of the LS group roughly returned to the preoperative level, while the recovery of the OS group was relatively worse; 12 months following the surgery, the quality of life of both groups significantly improved, and that of the LS group was better than the OS group. Additionally, the Kaplan–Meier analysis indicated that the 3-year survival rate of patients underwent LS was 81.67%, and that of the OS was 80.00%, with no significant difference between the two cohorts. Therefore, LS of CRC can achieve the same long-term efficacy as OS of CRC. Consistently, Cui et al. suggested that there is no noteworthy difference in the long-term quality of life between LS and OS for CRC patients (26).

This study has several limitations. First, it was a single-center, retrospective, non-randomized analysis, and the allocation to LS or OS was based on clinical judgment and patient preference, so residual selection bias cannot be completely excluded despite comparable baseline characteristics between the two groups. Second, the sample size was relatively modest and the follow-up period was limited to 3 years, which may reduce the power to detect small differences in long-term oncological outcomes. Third, because of the retrospective design, information on neoadjuvant chemoradiotherapy for rectal cancer was not reliably captured in the dataset used for this analysis and could not be evaluated, which may have influenced the indications for Miles surgery. Fouth, quality-of-life outcomes were based on self-reported GIQLI scores collected without strict blinding to the surgical approach, so some degree of reporting or assessment bias cannot be ruled out. Fifth, detailed data on postoperative tumor recurrence (local or distant) were not consistently available because recurrence was not systematically recorded during follow-up; therefore, we were unable to compare disease-free survival between the two groups and restricted our long-term analysis to overall survival. Therefore, larger, multicenter prospective studies with longer follow-up are needed to confirm and extend our findings.

## Conclusion

LS for CRC patients is highly effective, alleviating inflammatory and immune stress responses in patients, lowering the incidence of postoperative complications, improving the quality of life of patients, and having a long-term efficacy comparable to OS.

## Data Availability

The original contributions presented in the study are included in the article/Supplementary Material, further inquiries can be directed to the corresponding author.
